# Zygomatic Implant-Supported Prosthetic Rehabilitation of a Patient with Post-COVID-19 Mucormycosis and Maxillectomy

**DOI:** 10.1155/2024/8193822

**Published:** 2024-05-10

**Authors:** Rewa Kawade, Seema Sathe, Anjali Bhoyar, Bhushan Mundada, Neeraj Chachediya, Aditee Apte

**Affiliations:** ^1^Department of Prosthodontics & Crown and Bridge, Sharad Pawar Dental College and Hospital, Datta Meghe Institute of Higher Education and Research (DMIHER), Sawangi(M), Wardha, India; ^2^Department of Oral and Maxillofacial Surgery, Sharad Pawar Dental College and Hospital, Datta Meghe Institute of Higher Education and Research (DMIHER), Sawangi(M), Wardha, India

## Abstract

Mucormycosis has been prevalent in the past few years with the COVID-19 pandemic largely affecting the maxilla due to its proximity to the respiratory corridor. Resection of the maxillary dentition along with the maxillary bone itself has a huge psychosocial impact on the patient. When seeking prosthetic rehabilitation, poor esthetics, difficulty in mastication, and social embarrassment are the patients' concerns. Hence, it becomes great responsibility for the clinician to restore the patient's natural teeth while causing him/her minimum discomfort. This article describes such a case of post-COVID-19 mucormycosis that has been provisionally restored by the means of all-on-four zygomatic implants.

## 1. Introduction

Edentulism is a common concern for patients treated for mucormycosis followed by COVID-19. Most of these patients undergo varying degrees of maxillary resection that cause edentulism of varying severity. The maxillary teeth and the underlying residual alveolar ridge are lost, leading to severely hampered esthetics and mastication that eventually affects the patient's health, i.e., both psychological and physical.

Mucormycosis is an opportunistic fungal infection that occurs in the presence of an existing systemic disease. The microorganisms spread from the respiratory mucosa to the maxillary antrum, nasal and paranasal sinuses, and the oral cavity. This infection eventually leads to thrombosis of blood vessels and necrosis of tissues leaving aggressive surgical debridement as the treatment of choice. Maxillary tissue left is usually with a defect or only soft tissue. Contingent upon the augmentation of the necrosed tissue, the patient would need to go through maxillectomies of various amounts. The classification for the maxillary defects proposed by Aramany [1] and Brown et al. [2] is the most broadly perceived for the reason.

Rehabilitation of defects of the maxilla poses a challenge to the prosthodontist due to the poor foundation available. In such cases, placement of endosseous implants provides a stable foundation for the future prosthesis rendering sufficient retention and patient acceptability [3]. The ideal sites for the placement of implants for patients with maxillary defects are the premaxillary segment and the maxillary tuberosity [3]. However, in the absence of bone in these sites, the pterygoid and zygomatic bones can be utilized [3]. This provides a graft-less approach for the placement of implants [4]. Bedrossian et al. and Davo et al. suggested that the number of implants placed can be either two zygomatic with two axial implants [5] or two to three zygomatic implants in each bone for maxillary rehabilitation [6].

The temporization of these implants can be done using the denture conversion technique as proposed by Misch [7]. Zygomatic implants are advised to be loaded immediately [8–10] although intermediate loading is acceptable. The definitive prosthesis is then fabricated after 3 months [11]. This case report presents a method of rehabilitation of a patient with total maxillectomy without a defect using zygomatic implants and temporization with implant-supported prosthesis using temporary metal cylinders.

## 2. Case Presentation

**Figure 1:**
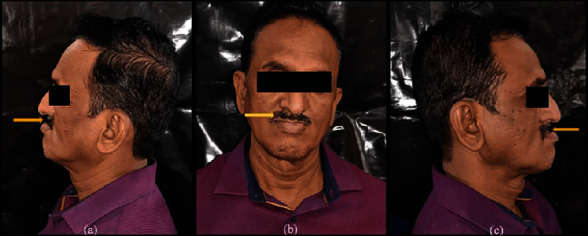
Extraoral preoperative photograph: (a) left lateral view; (b) frontal view; (c) right lateral view showing sunken upper lip represented by yellow arrow.

**Figure 2:**
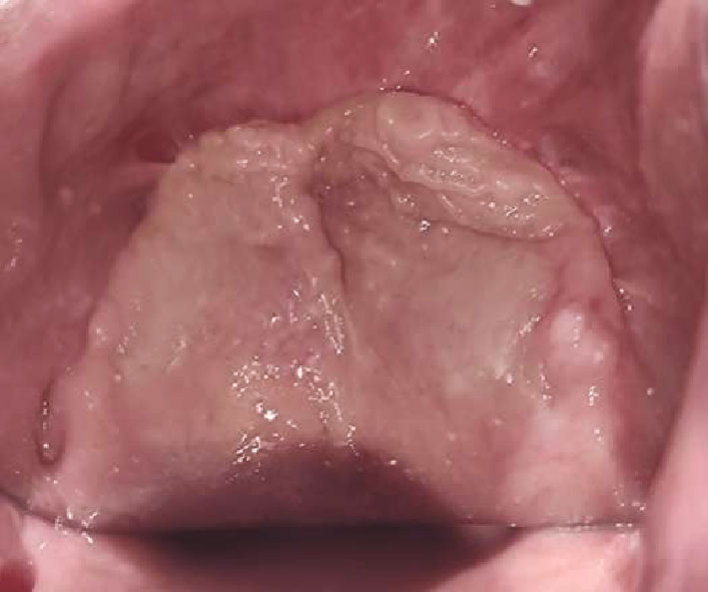
Completely edentulous maxillary arch.

**Figure 3:**
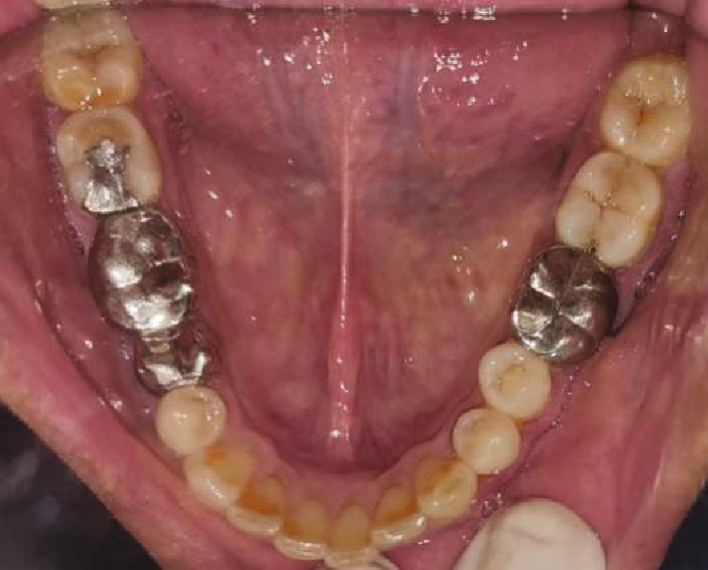
Mandibular arch.

A 49-year-old male patient reported to the Department of Prosthodontics, Sharad Pawar Dental College and Hospital, Sawangi, Wardha, with the chief complaint of difficulty in mastication due to missing teeth in the upper jaw. His medical history revealed that he tested positive for COVID-19 one year back. The patient was detected with mucormycosis secondary to COVID-19 for which he underwent bilateral inferior level maxillectomy. Therefore the patient's main concern was the loss of lip fullness as shown in Figures 1(a)–1(c). Intraoral examination revealed an edentulous maxillary arch (Figure 2) and a dentulous mandibular arch (Figure 3). Maxillary ridge was flabby and irregular with a square arch form, and metal crowns were seen with 35, 36, and 46 in the mandibular arch.

The flabby tissues and absence of any bony foundation would result in poor retention properties of the removable prosthesis. Hence, an option of the implant-supported supported prosthesis was given to the patient. The CT scan of the patient revealed the absence of any bone present in the maxilla with only the zygomatic bone in a sufficient amount; therefore, it was decided to take the anchorage for the implant in the zygomatic bone. The defect was classified according to Brown's classification as a bilateral class 2 with horizontal component c.

**Figure 4:**
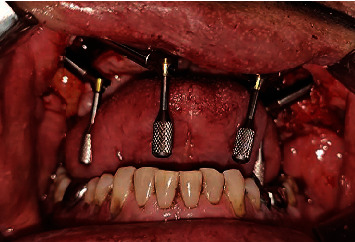
Angled abutments attached to the zygomatic implants.

**Figure 5:**
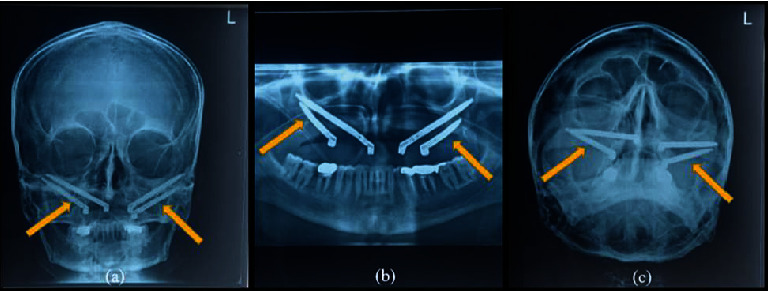
Postsurgical radiographs of the patient: (a) anteroposterior view; (b) orthopantomogram; (c) submentovertex radiographs showing zygomatic implants in place.

Diagnostic impressions were made for maxillary and mandibular arches with irreversible hydrocolloid impression material (Dentsply Vignette chromatic), and diagnostic casts were obtained. The patient was prescribed amoxicillin 1 hour prior to the surgery. Surgery was carried out under general anesthesia. The incision was made with cautery, and the tissues reflected on both sides. Four zygomatic implants (Noris Zygo™) measuring each 37.5 mm in 15 with 60-degree abutment (Angulated Multi-unit NM-A7160), 57.5 mm in 12 with 60-degree abutment (Angulated Multi-unit NM-A7160), 37.5 mm in 25 with 60-degree abutment (Angulated Multi-unit NM-A7160), and 55 mm in 23 with 45-degree abutment (Angulated Multi-unit NM-A7146) were placed (Figure 4). Healing abutments (Healing Cap NM-H7102) were attached, and suturing was done using absorbable sutures (vicryl). Postsurgical radiographs of the patient were taken (Figures 5(a)–5(c)).

**Figure 6:**
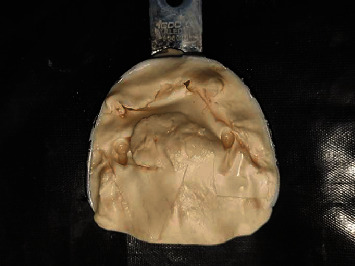
Hydrocolloid impression made with healing caps postsurgical after placement of implants.

**Figure 7:**
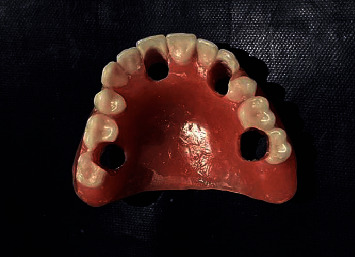
Maxillary denture with holes prepared in the sites of the healing caps.

The rubber dam was placed on the healing abutments, and another impression was made with irreversible hydrocolloid impression material (Figure 6). A temporary record base was fabricated (DPI-RR Cold Cure), and jaw relation was recorded using Niswonger's method (vertical jaw relation) and interocclusal check record method for centric jaw relation. Try-in was done, and the denture was fabricated. Since the denture was fabricated on the impressions made on the healing caps, the tentative positions for the temporary cylinders were already recorded. Holes were made in the same positions for the temporary cylinders (Figure 7).

**Figure 8:**
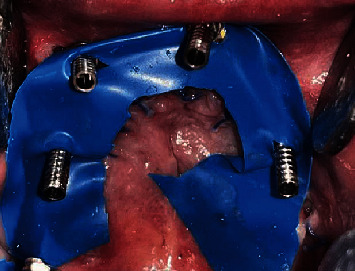
Intraorally attached temporary cylinders.

**Figure 9:**
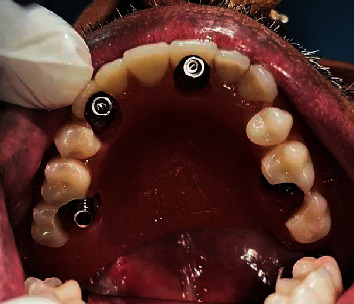
Denture placed intraorally on temporary cylinders.

**Figure 10:**
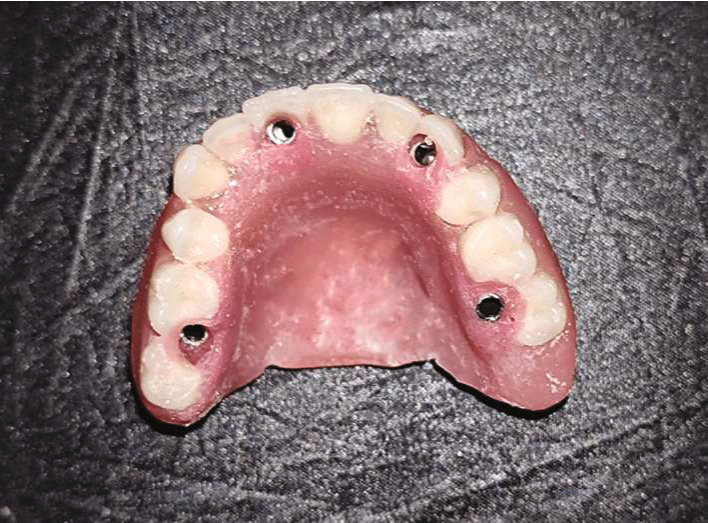
Maxillary denture with temporary cylinders picked up.

The temporary cylinders (Universal Abutments NM-T7121) were then screwed to the abutments intraorally (Figure 8), and the denture was tried to verify the position of the cylinders in dentures so that there was 2 mm space available for the autopolymerizing acrylic resin on all sides (Figure 9). Adjustments were done on the denture for the same, and occlusion was obtained in canine guidance. Temporary cylinders were then picked up in the denture (Figure 10).

**Figure 11:**
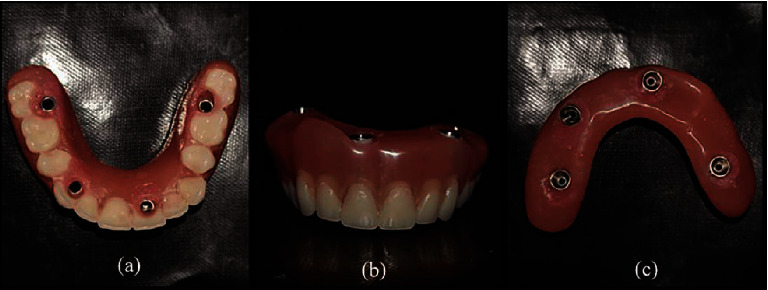
Finished maxillary prosthesis: (a) occlusal view; (b) frontal view; (c) intaglio surface.

**Figure 12:**
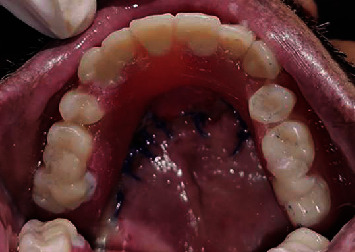
Prosthesis inserted intraorally (occlusal view).

**Figure 13:**
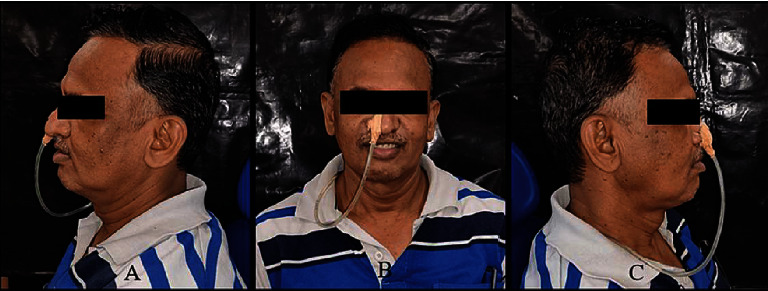
Extraoral photographs of patient with inserted final prosthesis: (A) left lateral view; (B) frontal view; (C) right lateral view.

The denture was then modified, and flanges and palate were removed, finished polished, and inserted (Figures 11(a)–11(c)). Occlusion was verified again by selective grinding of the teeth, and the access openings of screws were blocked with Teflon tape and sealed with composite (Figure 12). The patient was satisfied with the appearance (Figure 13). The patient was kept on follow-up every 7 days for verification of phonetics, occlusion, and comfort.

## 3. Discussion

Loss of the maxillary jaw leads to ineffective speech and mastication along with poor social interactions due to reduced esthetics. Placement of a prosthesis in such a patient poses a challenge to the prosthodontist due to poor foundation and loss of retention. Utilization of the underlying undercuts can prove to be effective to gain retention when there is some amount of bone remaining. Reconstruction of the maxilla in cases of severely resorbed ridges can be done using grafting procedures [12], but it makes the treatment longer with the need for an additional surgical procedure [5].

Zygomatic implants can become a blessing for cases in which no bone remains postresection of the affected region. The placement of two axial implants in the premaxillary region is advocated for the success of implants [5]. In addition, the placement of two to three zygomatic implants can be done in each without the placement of axial implants [6]. Temporization for this case was done in an innovative way. The conventional fabrication steps involve the use of surgical guides. However, in cases with unpredictable bone and the affordability of the patient, the placement of an implant based on the surgeon's skill can prove effective. The impression for this case was made after the placement of healing caps with rubber dam used as a shield to protect the underlying tissues. This serves both purposes—accuracy and predictability of the abutment position in the prosthesis.

This technique can be used effectively for fabrication of temporary prosthesis in patients with zygomatic implant-supported prosthesis and all on four concept as well. The survival rate and complications of the zygomatic implants have been studied over years to find that they have a high cumulative survival rate of 96.7% as reported by Chrcanovic and Abreu [13]. Pterygoid implants or patient-specific implants can be selected for cases depending on bone availability. Temporization with a fixed implant-supported prosthesis improves the confidence of the patient to return to his daily routine.

## 4. Conclusion

This case report describes the rehabilitation mucormycosis-treated patient with an implant-supported prosthesis. The method of denture conversion is modified in this patient as the denture was fabricated on impressions made after placement of the implants. This can be helpful when the bone availability and quality are questionable and guided implant surgery is not feasible. Fabrication and conversion of the denture into a fixed prosthesis were simplified, and the patient was provided with his missing piece of confidence through an esthetic pleasing smile.
